# Molecular detection and genetic characterization of Wenzhou virus in rodents in Guangzhou, China

**DOI:** 10.1186/s12917-021-03009-2

**Published:** 2021-09-08

**Authors:** Nina Wang, Lichao Yang, Guohui Li, Xu Zhang, Jianwei Shao, Jun Ma, Shouyi Chen, Quan Liu

**Affiliations:** 1 School of Life Sciences and Engineering, Foshan University, 440605 Foshan, Guangdong Province China; 2 Guangzhou Center for Disease Control and Prevention, 510440 Guangzhou, Guangdong Province China

**Keywords:** Wenzhou virus, mammarenavirus, rodents, phylogenetic analysis, Guangzhou, China

## Abstract

**Background:**

Wenzhou virus (WENV), a newly discovered mammarenavirus in rodents, is associated with fever and respiratory symptoms in humans. This study was aimed to detect and characterize the emerging virus in rodents in Guangzhou, China.

**Results:**

A total of 100 small mammals, including 70 *Rattus norvegicus*, 22 *Suncus murinus*, 4 *Bandicota indica*, 3 *Rattus flavipectus*, and 1 *Rattus losea*, were captured in Guangzhou, and their brain tissues were collected and pooled for metagenomic analysis, which generated several contigs targeting the genome of WENV. Two *R. norvegicus* (2.9%) were further confirmed to be infected with WENV by RT-PCR. The complete genome (RnGZ37-2018 and RnGZ40-2018) shared 85.1–88.9% nt and 83.2–96.3% aa sequence identities to the Cambodian strains that have been shown to be associated with human disease. Phylogenetic analysis showed that all identified WENV could be grouped into four different lineages, and the two Guangzhou strains formed an independent clade. We also analyzed the potential recombinant events occurring in WENV strains.

**Conclusions:**

Our study showed a high genetic diversity of WENV strains in China, emphasizing the relevance of surveillance of this emerging mammarenavirus in both natural reservoirs and humans.

**Supplementary Information:**

The online version contains supplementary material available at 10.1186/s12917-021-03009-2.

## Background

Mammarenaviruses, belonging to the *Mammarenavirus* genus in the *Arenaviridae* family, include several members responsible for severe hemorrhagic fever diseases. Of them, Lassa virus (LASV) may cause an approximate 100,000-300,000 case and 5000 deaths annually in Western Africa (Lassa Fever CDC, 2019, https://www.cdc.gov/vhf/lassa/index.html), while Junín virus (JUNV), Guanarito virus (GTOV), Machupo virus (MACV), and Sabía virus (SABV) can cause fatal hemorrhagic fever diseases in Latin America [1]. In Asia, the known mammarenaviruses are lymphacytic choriomeningitis virus (LCMV), Wenzhou virus (WENV), Loei River virus (LORV), Ryukyo virus (RVKV), Xingyi and Lijiang viruses. LCMV infection can lead to various clinical manifestations, ranging from asymptomatic to influenza-like illness, aseptic meningitis, or meningoencephalitis. LCMV is also associated with congenital microencephaly in pregnant women [2, 3]. WENV, first discovered in 2014 in Wenzhou, Zhejiang Province, China [4], has been shown to be related to fever and respiratory symptoms in humans [5]. LORV was reported in 2016 from Thailand, and RVKV, Xingyi and Lijiang viruses were all found in China during 2018–2019, whose relevance to public health remains unclear [5–7].

Mammarenaviruses are negative-sense single-stranded RNA viruses that include the small (S) and large (L) genomic segments. The S segment encodes an envelope glycoprotein precursor (GPC) and a nucleoprotein (NP), and the L segment encodes a RNA-dependent RNA polymerase (RdRp) and a zinc binding matrix protein (ZP) [8]. Rodents are considered as the primary reservoir for mammarenaviruses, with several exceptions. For example, Tacaribe virus (TCRV) has been isolated from fruit-eating bats (*Artibeus jamaicensis*) and ticks, LCMV has been isolated from ticks, and WENV has also been detected in shrews [4, 9–11]. Based on genetic and geographic relationships, mammarenaviruses are divided into two monophyletic groups, including the New World (NW) and the Old World (OW). The NW arenaviruses, including JUNV, GTOV, MACV, SABV, WWAV, and TCRV, are geographically confined to Americas. While the OW arenaviruses are generally distributed in Africa (LASV) and Asia (WENV, LORV, RVKV, Xingyi and Lijiang virus) [8]. The high genetic diversity has been shown in the NW and OW arenaviruses [12, 13], and WENVs from different geographic area have formed independent clades [7, 14].

Diverse zoonotic pathogens (i.e., hantaviruses, hepatitis viruses, *leptospira* spp., *bartonella* spp.) have been reported in rodents in Guangdong Province [15, 16]. However, little is known about WENV circulation status in Guangzhou. Herein, we attempt to detect and characterize of WENV, which may contribute to understanding the evolution of WENV.

## Results

### Detection of WENV in rodents in Guangzhou

**Fig. 1:**
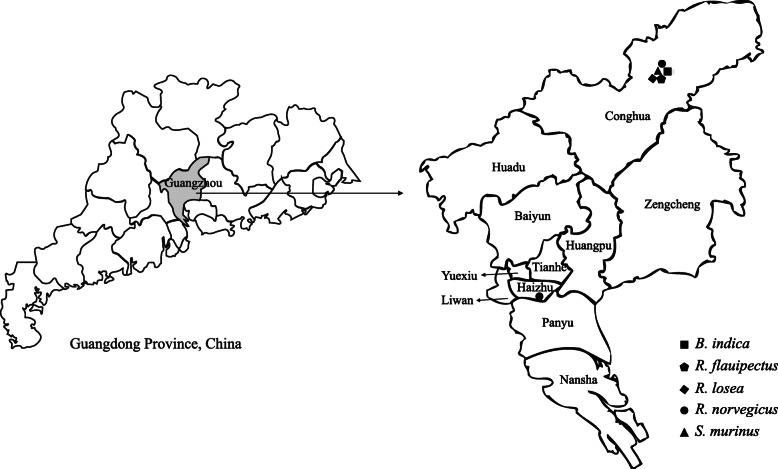
The sampling sites in Guangzhou, Guangdong Province, China. This map was drawn by us for this study, and plotted by combination of Surfer software version 4 (Golden software, USA) and Adobe illustrator CS6 (Adobe Systems, USA)

In 2018, a total of 100 rodent samples were collected from Conghua and Haizhu Districts in Guangzhou, Guangdong Province, China (Fig. 1). The rodents included 3 genera and 5 species, including 70 *Rattus norvegicus*, 22 *Suncus murinus*, 4 *Bandicota indica*, 3 *Rattus flavipectus and 1 Rattus losea* (Table 1). Brain tissues of each rodent were collected for viral metagenomic analysis, which produced 2,722 contigs, with an average length of 211 nt. There were 12 (0.44%) contigs annotated to the L and S segment of WENV, with 88.5–92.9% and 90.8–95.8% nt identities, respectively (Table S1). Semi-nested RT-PCR targeting the 290 nt fragment of S segment was performed to screen WENV infection in all collected samples, showing that two *R. norvegicus* (2/70, 2.9%) in Conghua District were positive for WENV. One of the two *R. norvegicus* was male, adult; the other was female, juvenile. No WENV infection was detected in *B. indica*, *R. flavipectus*, *R. losea*, or *S. murinus*.

**Table 1:** Detection of WENV in rodents captured in Guangzhou, China

Sampling Sites	Species	Sex		Weight		Age Category		P/D^a^
		**Female**	**Male**	**> 200 g**	**≤ 200 g**	**Juvenile**	**Adult**	
**Conghua District**	*Bandicota. indica*	0	4	1	3	0	4	0/4
	*Rattus. flauipectus*	2	1	0	3	0	3	0/3
	*Rattus. losea*	1	0	1	0	0	1	0/1
	*Rattus. norvegicus*	9	11	10	10	5	15	2/20
	*Suncus. murinus*	11	11	0	22	1	21	0/22
**Haizhu District**	*Rattus. norvegicus*	19	31	37	13	10	40	0/50
**Total**		42	58	49	51	16	84	2/100

^a^*P* positive numbers; *D* detected sample numbers

## Genetic analysis of WENV

The complete S and L segments were recovered from the two *R. norvegicus* samples (RnGZ37-2018 and RnGZ40-2018), which comprised the S (3,350 nt) and L (7,118 nt) segments, each containing two open reading frames (ORFs) in an ambisense organization with an intergenic region (IGR). L segment encoded a ZP of 276 nt (91 aa) and a RdRp of 6672 nt (2,223 aa), connected by an IGR of 122 nt. S segment encoded a NP of 1704 nt (567 aa) and a GPC of 1479 nt (492 aa), connected by an IGR of 62 nt (Table S4). Interestingly, the 122 nt IGR of the L segment from WENV in this study was longer than other WENV isolates (~ 119 nt) [7].

Sequence comparison revealed that RnGZ37-2018 and RnGZ40-2018 were closely related with each other, with 93.5–98.6% nt and 95.1–97.6% aa identities, and had 83.5–93.3% nt and 84.2–91.5% identity for the S and L segments in comparison with other WENV strains, respectively (Table 2). The nt and aa identity for RdRp, ZP, NP and GPC were also shown in Table 2. RnGZ37-2018 and RnGZ40-2018 were most closely related to the 9–24 strain, with 90.9–93.9% nt and 93.3–98.0% aa identity. Notably, both RnGZ37-2018 and RnGZ40-2018 were also closely related to the Cambodian strains, which were identified in flu-like patients, with 85.1–88.9% nt and 83.2–96.3% aa identity [5].

**Table 2:** Genome and ORFs comparisons between RnGZ37-2018, RnGZ40-2018 and other representative WENV strains

Strains	Segment/ORFs	% nt/aa sequence identities within WENV^a^										
		**RnGZ37-2018**	**Cambodian C617**	**Cambodian C649**	**9–24**	**YCB1**	**Rn366**	**Rn242**	**MYR039**	**G107**	**PL/DK**	**RnYM3-2016**
**RnGZ37-2018**	L	/	87.5	87.3	91.5	84.4	87.9	88.9	86.8	87.5	89.2	84.6
	RdRp	/	87.7/91.9	87.6/91.7	91.1/93.4	84.7/89.6	89.5/93.6	89.3/93.3	87.2/91.7	87.6/91.7	89.5/93.1	84.7/89.8
	ZP	/	86.2/83.2	86.6/94.1	91.7/97.0	83.0/93.1	89.9/91.1	81.2/85.1	86.2/89.1	88.4/90.1	87.3/89.1	85.5/94.1
	S	/	86.7	86.6	93.3	85.6	88.5	87.7	87.1	88.2	86.4	83.6
	NP	/	88.6/95.4	88.7/95.6	93.9/96.6	86.7/94.7	89.1/95.6	88.8/94.5	88.7/94.9	89.6/93.8	88.4/93.3	86.2/94.5
	GPC	/	85.5/93.1	85.2/92.7	90.9/94.3	84.8/93.1	86.7/91.9	88.2/93.5	85.5/93.3	87.4/94.9	86.9/92.1	84.6/92.9
**RnGZ40-2018**	L	96.0	87.3	87.1	91.4	84.2	87.6	88.7	86.7	87.3	88.9	84.4
	RdRp	95.8/97.6	87.5/91.7	87.4/91.5	90.9/93.3	84.4/89.4	89.1/93.4	89.0/93.1	86.9/91.4	87.4/91.7	89.1/92.8	84.5/89.3
	ZP	98.6/97.0	86.6/84.2	87.0/95.0	92.4/98.0	83.3/94.1	90.2/92.1	81.5/86.1	86.6/90.1	88.8/91.1	87.7/90.1	85.9/95.0
	S	95.1	86.9	86.7	92.7	85.2	88.7	87.7	86.8	88.1	86.8	83.5
	NP	96.0/97.2	88.8/96.1	88.9/96.3	93.6/97.4	86.3/95.1	90.1/96.6	89.6/95.8	88.6/95.2	89.8/94.9	89.3/94.5	86.2/95.1
	GPC	93.5/95.1	85.3/93.1	85.1/92.7	91.8/93.7	85.2/92.5	87.5/91.5	89.2/92.9	85.9/93.1	87.8/93.5	87.1/91.5	84.7/91.9

^a^ The reference sequences accession number and sampling sites were indicated in Table S3

### Phylogenetic analysis of WENV

**Fig. 2:**
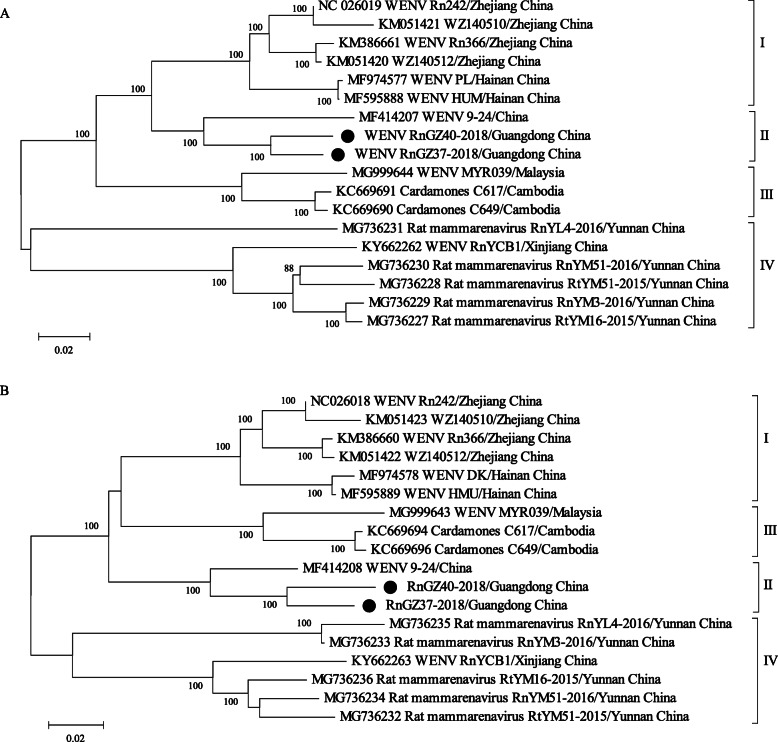
The maximum likelihood trees based on the full-length genomic sequences of WENV. The phylogenetic trees of the L (**A**) and S (**B**) segments of WENV were constructed using ML methods based on GTR model with gamma distribution and invariant sites, and tested with 1000 bootstrap replicates. Sequence were identified by the GenBank accession number and the strain name, followed by their origin. The newly identified WENV variants in Guangzhou were indicated with black circles

Phylogenetic analysis based on the full-length of the S and L segments showed that WENVs formed a monophyletic clade distinct from other OW arenaviruses [4, 14]. RnGZ37-2018 and RnGZ40-2018 were clustered together and formed an independent clade within WENV (Fig. 2). According to the evolutionary relationships, both S and L segment of all WENV strains (except WENV G107) could be grouped into 4 different lineages. Based on the S segment tree, lineage I included WENV strains from Zhejiang and Hainan; the novel WENVs identified in Guangzhou were clustered in lineage II; lineage III contained the strains identified in patients in Cambodia; and lineage IV was comprised the virus isolated in Yunnan and Xinjiang (Fig. 2A). Phylogenetic topology of L segment was the same as the S segment (Fig. 2B). Genetic diversity and protein differences within and between the viral lineages were shown in Table 3. Genetic distance of within or between lineages on the L segment was like the S segment, with the mean *p*-distance ranging from 0.07 to 0.75. The aa divergence of RdRp, ZP, GPC, and NP were 0.33, 0.27, 0.25, and 0.09, respectively, suggesting that NP is highly conserved in comparison with other proteins.

**Table 3:** Genetic and protein diversity of WENV strains

Lineages	Mean p-distance*					
	**L, nt**	**S, nt**	**RdRp, aa**	**ZP, aa**	**GPC, aa**	**NP, aa**
Overall	0.70	0.67	0.33	0.27	0.25	0.09
Within lineages I	0.56	0.72	0.55	0.54	0.52	0.04
Within lineages II	0.49	0.07	0.16	0.00	0.06	0.15
Within lineages III	0.75	0.51	0.03	0.08	0.04	0.02
Within lineages IV	0.74	0.70	0.21	0.09	0.05	0.03
Between lineages I and II	0.67	0.64	0.41	0.35	0.36	0.17
Between lineages I and III	0.74	0.74	0.40	0.37	0.35	0.06
Between lineages I and IV	0.74	0.69	0.46	0.38	0.36	0.06
Between lineages II and III	0.74	0.75	0.13	0.07	0.07	0.16
Between lineages II and IV	0.74	0.64	0.21	0.09	0.08	0.16
Between lineages III and IV	0.71	0.68	0.17	0.12	0.07	0.05

*p-distance is the number of nucleotide substitutions divided by the number of nucleotides. Pairwise genetic distances (uncorrected *p* values) were calculated using Mega X by eliminating all positions containing alignment gaps and missing data

### Recombination analysis of WENV

Both RDP4 and Simplot methods were used to analyze potential recombination events of WENVs. No obvious recombination events were found in RnGZ37-2018 and RnGZ40-2018 (Figure S1 and Table S5). Interestingly, the potential recombinant events in WENV isolates were found using RDP4 (*p* < 0.05 and the RDP recombination consensus score > 0.6), showing that the potential recombinant events occurred in the S segment of RnYM3-2016 between RtYM51-2015 and RnYL4-2016, and the Rn242 between DK and WZ140510, also as in the L segment of WZ140510 between WZ140512 and an unknown isolate (Table S5).

## Discussion

WENV was first identified in rodents and shrews by nested RT-PCR using the universal primers of arenaviruses in Wenzhou, Zhejiang Province in China in 2014, where *R. flavipectus* (15.4%), *R. losea* (11.8%), *R. norvegicus* (17.1%), *R. rattus* (75.0%), *Niviventer niviventer* (1.9%), and *S. murinus* (4.4%) were tested positive [4]. Since then, the virus has been found in *R. norvegicus* in Shandong, Hainan and Xinjiang Provinces [7, 17, 18], also found in *R. losea* in Hunan Province [6]. In Shenzhen, Guangdong Province, WENV was found in *R. norvegicus* and *S. murinus* with an infection rate of 6.7% and 0.5%, respectively [15]. In Yunnan Province, WENV has been found in *R. nitidus*, *R. norvegicus*, *R. tanezumi*, and *Tupaia belangeri*, with an infection rate of 8.3%, 17.4%, 1.5%, and 10%, respectively [14]. WENV has also been detected in Southeast Asia, including in 14.6% *R. exulans* and 21.1% *R. norvegicus* from one Cambodian province, whilst the closely related LORV was found in 25% *B. indica*, 28.6% *Bandicota savilei* and 1.9% *Niviventer fulvescens* in the Thai province of Loei [5]. In this study, two WENV isolates RnGZ37-2018 and RnGZ40-2018 were identified in the brain tissues of *R. norvegicus* (2.9%) but none was found in other rodent species in Guangzhou (Table 1), probably owing to the limited numbers and the brain tissue used. *S. murinus* was the carrier of WENV with a rate varying from 0.5–4.4%, and our results were consistent with previous studies showing a rate of 0.5% in Shenzhen [4, 15]. WENV has been shown to replicate in brain tissue in an experimental rat model, with higher viral loads in liver, lung and spleen [5, 7], which may explain the relatively lower rate in the brain tissues [4, 14, 15].

*R. norvegicus* has been considered as the host of WENV, which is also infected with LCMV [19, 20]. LCMV has been divided into 3 and 4 lineages based on L and S segment, respectively, and shows no clear correlation between the genetic diversity and geographic locations [10, 12]. To date, WENV has been reported only in Asia, and clusters together according to their geographic distribution [7, 14]. Remarkably, though Shenzhen and Guangzhou are over 100 kilometers away, the WENV forms two different sister clades (Figure S2), showing a high genetic diversity of WENV, which is consistent with previous study [21]. The 9–24 strain was most closely related to the Guangzhou strains but the geographic origin of this strain is unknown. Thus, the geographical relationship between the 9–24 strain and the Guangzhou strains was not discussed in this study. Interestingly, the IGR of L segment of WENV identified in Guangzhou was longer than most known WENV (Table S4), and substitution or deletion of IGR in L segment in LASV and MACV can reduce viral titer compared to wide type [22, 23]. However, the effect of the longer IGR on viral replication needs further study. Recombination and reassortment may contribute to forming high genetic diversity in mammarenaviruses [24, 25]. The first evidence of recombination in S segments within WENV was sampled in Yunnan Province with RnYM3-2016 isolates [14]. The potential recombinant event in S segment with Rn242 (Zhejiang Province) and L segment with WZ140510 (Zhejiang Province) were also observed (Table S5). These data have shown a complex evolutionary history of WENVs.

WENV infection may cause diffuse pneumonia in the experimental rat models [5, 7], and also cause a small cranial cavity in *R. exulans*, suggesting microcephaly as observed in LCMV [26, 27]. Moreover, WENV has been found seropositive (17.4%) in dengue-like/influenza-like patients and healthy individuals in Southeastern Asia, and 4.6% in healthy individuals in China [5, 28]. It is likely to show that the impact of WENV infections on public health may be underestimated, as the symptoms are mild and similar to those of respiratory diseases.

## Conclusion

New genotypes of WENV in *Rattus norvegicus* from Guangzhou of China were identified. The results showed that WENVs from Guangzhou clustered together, and they were closely related to the Cambodian strains associated with human disease, and WENV from Guangzhou also showed highly genetic diversity in China. These findings highlight the importance of surveillance of WENV in both natural reservoirs and humans.

## Methods

### Specimen collection

In 2018, wild rodents were trapped using cages in residential areas in two districts (Conghua and Haizhu) of Guangzhou (Fig. 1). Trap cages were placed next to garbage cans and human settlements. The rodents were euthanized by the inhalation of carbon dioxide by trained personnel with appropriate technique, equipment, and agents [29]. The trapped rodent transferred to euthanasia chamber connected with a CO_2_ tank which was equipped with a pressure regulator and flowmeter. Exposed to 30% CO_2_ for at least 3 minutes or longer at a flow rate of 3.4 L/min until rodents became unconsciousness, even stopping breathing. Leaving rodents exposed to 70% CO_2_ for an additional 2 minutes at a flow rate of 7.9 L/min and turning off the CO_2_. Death was ascertained by absence of movement, cardiac and respiratory arrest, fixed and dilated pupils. Death was further confirmed by decapitation (> 200 g) or cervical dislocation (≤ 200 g). The blood and organs were collected by necropsy, then stored at -80℃. Rodents species were classified by morphology and subsequently confirmed by sequencing the mitochondria cytochrome *b* gene [30]. The species, sex, weight, and age category (juvenile or adult) were recorded in Table 1.

### Viral metagenomic analysis

The rodent brain tissues (~ 50 mg) were homogenized with Qiagen tissue lyser II (Qiagen), and used for viral metagenomic analysis as previously described [31, 32]. Briefly, the viral nucleic acids were extracted and reverse transcribed with anchored random primers. The double-strand cDNA was amplified using sequence-independent, single-primer amplification, then PCR products were purified. The equal amount of the purified PCR products from 100 samples were pooled together and sent to the Beijing Genome Institute (BGI, Shenzhen, China) for high-throughput sequencing. All generated sequences were subjected to BLASTx and BLASTn search against the viral reference database for further analysis.

### WENV screening and complete genome recovery

Based on the WENV-like contigs from the metagenomic analysis, we designed the semi-nested RT-PCR primers that targeted a 290 nt fragment of the S segment (F1748: 5'-TTCTTCTTTTCAACAACCAC-3'; R1-2055: 5'-GAGCCAACAGACGCCAAG-3'; R2-2037: 5'-CAGTTCAAGCAAGACTCT-3'). Total RNA was extracted with MiniBest viral RNA/DNA extraction kit (TaKaRa), and reverse transcription was conducted with the 1st cDNA synthesis kit (TaKaRa). cDNA was amplified by using the ExTaq (TaKaRa) with the following PCR parameters: 94℃ for 5 min, followed by 35 cycles of 94℃ for 35 s, 51℃ for 35 s, 72℃ for 25 s, and a final elongation step at 72℃ for 10 min. The PCR products were analyzed on gel electrophoresis, and positive samples were further sequenced.

To get complete genomic sequences, overlapping primers (Table S2) that covered the terminal ends were designed according to the WENV-like contigs and reference sequences (GenBank accession numbers MF414208 and MF414207). The amplicons were cloned to the pMD18-T vector (TaKaRa), and at least two clones for each PCR products were sequenced to obtain a consensus sequence. The resulting sequences were assembled into the complete genome using SeqMan in DNASTAR.

### Genomic and phylogenetic analysis

The complete genome sequences of WENV representative strains were retrieved from GenBank and listed in Table S3. Pairwise sequence alignment was performed using DNASTAR [33]. Phylogenetic analysis was conducted with the maximum likelihood (ML) method based on the general time reversible model with gamma distribution and invariant sites, and tested with 1000 bootstrap replicates in MEGA X program, also the genetic distance was tested by *p*-distance with transitions and transversions method [34]. The recombination of S and L segments was analyzed by Recombination Detection Program version 4 (RDP4) and Simplot [35, 36].

## Supplementary Information

**Additional file 1:** **Supplementary Table 1. **The contigs annotated to WENV. **Supplementary Table 2. **The primers used for amplifying the complete genome of WENV. **Supplementary Table 3. **The reference sequences used in this study. **Supplementary Table 4. **The genome organization of Wenzhou virus strains identified in Guangzhou. **Supplementary Table 5. **WENV recombination events detected using the RDP package. **Supplementary Fig. 1. **The recombination analysis of RnGZ37-2018 and RnGZ40-2018 by similarity plots. The recombination events were analyzed based on the full-length of L/S from RnGZ37-2018, RnGZ40-2018 and other representative WENV strains. RnGZ37-2018 and RnGZ40-2018 were as query sequence. The recombination analysis: (A) S segment of RnGZ37-2018; (B) L segment of RnGZ37-2018; (C) S segment of RnGZ40-2018; (D) L segment of RnGZ40-2018. **Supplementary Fig. 2.** Phylogenetic analysis of the partial RdRp gene (589 bp) from WENV in Guangzhou and Shenzhen, China. The phylogenetic tree was performed using ML methods based on GTR model with gamma distribution and invariant sites, and evaluated with 1000 bootstrap replicates. Sequences were identified by the GenBank accession number and the strain name, followed by their origin. The partial sequences of WENV isolated from Shenzhen and Guangzhou were indicated.

## Data Availability

The data generated or analyzed in this study are included in the manuscript and the supplementary information files. The complete genome sequences obtained in this study have been submitted to GenBank with accession numbers: MW174777, MW174778, MW174779, and MW174780.

## Abbreviations

WENV: Wenzhou virus
NW: New World arenaviruses
OW: Old World arenaviruses
GPC: Glycoprotein precursor
NP: Nucleoprotein
RdRp: RNA-dependent RNA polymerase
ZP: Zinc binding matrix protein
ORF: Open reading frames
aa: Amino acid
nt: Nucleotide
IGR: Intergenic region
LASV: Lassa virus
LCMV: Lymphocytic choriomeningitis virus
JUNV: Junín virus
GTOV: Guanarito virus
MACV: Machupo virus
SABV: Sabía virus
LORV: Loei River virus
RVKV: Ryukyo virus
ML: Maximum likelihood method
RDP4: Recombination Detection Program version 4
*B. indica*: *Bandicota indica*; *R. norvegicus*
*Rattus norvegicus*: *R. rattus*:*Rattus rattus*
*R. flavipectus*: *Rattus flavipectus*
*R.losea*: *Rattus losea*
*R. exulans*: *Rattus exulans*
*R.nitidus*: *Rattus nitidus*
*R. tanezumi*: *Rattus tanezumi*
*S. murinus*: *Suncus murinus*
