# Supplementary material for: Molecular detection and genetic characterization of Wenzhou virus in rodents in Guangzhou, China
Source: BMC Vet Res. 2021 Sep 8;17:301. doi: 10.1186/s12917-021-03009-2 (PMC8424800; doi:10.1186/s12917-021-03009-2)
Supplement: Supplementary file 1 — Additional file 1: Supplementary Table 1. The contigs annotated to WENV. Supplementary Table 2. The primers used for amplifying the complete genome of WENV. Supplementary Table 3. The reference sequences used in this study. Supplementary Table 4. The genome organization of Wenzhou virus strains identified in Guangzhou. Supplementary Table 5. WENV recombination events detected using the RDP package. Supplementary Fig. 1. The recombination analysis of RnGZ37-2018 and RnGZ40-2018 by similarity plots. The recombination events were analyzed based on the full-length of L/S from RnGZ37-2018, RnGZ40-2018 and other representative WENV strains. RnGZ37-2018 and RnGZ40-2018 were as query sequence. The recombination analysis: (A) S segment of RnGZ37-2018; (B) L segment of RnGZ37-2018; (C) S segment of RnGZ40-2018; (D) L segment of RnGZ40-2018. Supplementary Fig. 2. Phylogenetic analysis of the partial RdRp gene (589 bp) from WENV in Guangzhou and Shenzhen, China. The phylogenetic tree was performed using ML methods based on GTR model with gamma distribution and invariant sites, and evaluated with 1000 bootstrap replicates. Sequences were identified by the GenBank accession number and the strain name, followed by their origin. The partial sequences of WENV isolated from Shenzhen and Guangzhou were indicated. [file 12917_2021_3009_MOESM1_ESM.pdf]

SUPPLEMENTARY INFORMATION

Supplementary Table 1. The contigs annotated to WENV

No. of contigs	Sequences	Length	Location	Identity (%)
1	CCGGAGCTCTGCAGATATCAATGGGTTAGGGC TAGACAGAGAAACCACTCCAAGAAAATGCCTG AGCGTGTGACGCCAACACTCAATCAAGTCTTC CAAGACATGATAAAAAACACTTATAAGAAAAT TGAATCAGCATTAGAATATGTTCACATGAAGC CTGAATCATATGGTGTC	177	9-24 (L) (1218-1373)	91.0
2	CGGAGCTCTGCAGATATCAGAAAGGATGGGGT TCAACCTACGATGAGTTATGAGCCCTCTCAGGC CAAAGAAATCCCATATGATATTGAGGTGATGG ACACACAGTCAGATGACGATTTCAGGGTTCTG TCGTCTCTTTGCTTAAGTATTGTGAACTCCATG AAAACCTCTTCAGTC	177	9-24 (L) (1417-1576)	91.3
3	CCGGAGCTCTGCAGATATCCCGTAGTTACGGA TCCGGCTTGCCGACTTTTCAAAGAATCTGAGGT AAAGGGTGACTTCATAGACCCTCTTGTTAGCTC AGGTTGTGCAACAGCCCTGGATTTAGCGAGCA ACAAGAGTGTTGTTGTCAATAAATTCACAAAA GATGGAAGG	171	9-24 (L) (2419-2548)	92.9
4	TTTGAGAATGCCCTGATAGCCATGAGGCTCTCT GTATCTCTCGCCCAGTTATCATATAGTCTAGAC CACAGTAAATGGGGACCCATGATGTGTCCTTTC TTATTTTTAATGTTGGTGCAAAACATAGATTTG AAATCACCCAGCGCTCTTGAGGGTATTAAGAG TGGATATCTGCAGAGCTCCGGC	186	Cardamones C617 (L) (3452-3619)	92.9
5	GCCGGAGCTCTGCAGATATCTGGGATTGAGTTT AGGAGGGTGGGGCCTGACTGGGAACCAGTGCC CCTTGTGTTGAAGAATGGCTACCAGGGGAATC CGACTGTTTAA	108	9-24 (L) (6240-6317)	88.5
6	GCCGGAGCTCTGCAGATATCTGGGACTGAATT CAGGAGGGTGGGGCCTGACTGGGAACCAGTGC CTCTTGTGCTGAAGGATGGTTACCTGTGGGAA GGGGAGAGAAAACTGGCACCACTAACGGCAG AGCTGCATACAGATGACCTAAAGGTGTTTATC CAAGAACTTCATGATGAA	177	9-24 (L) (6240-6397)	91.6
7	AATTCCCAAAGAGAGCAAACCGGCTGTGCCGC CTCCCTATAGGGCACCCGTCATCCCCGACACCA GTCATCTTGGCCCAACATTCTGCAAGAGTTGTT GGTTTGAGAGCAAGGGTCTTGTRGCRTGCTCCA ATCACTAYCTCTGCATGAGCTGCCTGACCCTTC TCCTCTCAGCCTCCGACCGCTGCCCCATCTGCA AGCTGCCTCTTCCCACGAAACTAGAGCTAAGC AGGACACCATCCGATATCTGCAGAGCTCCGGC	261	9-24 (L) (6836-7077)	92.6

8	CAAACCGGCTGTGCCGCCTCCCTATAGGGCAC<br>CCGTCATCCCCGACACCAGTCATCTTGGCCCAA<br>CATTCTGCAAGAGTTGTTGGTTTGAGAGCAAG<br>GGTCTTGTRGCRTGCTCCAATCACTAYCTCTGC<br>ATGAGCTGCCTGACYCTTCTCCTCTCAGCCTCC<br>GACCGCTGCCCCATCTGCAAGCTGCCTCTTCCC<br>ACGAAACTAGAGCTAAGCAGGACACCATCCGA<br>TATCTGCAGAGCTCCGGC	246	9-24 (L)<br>(6836-7062)	92.1
9	TTGATGAGCATCATCTCTACTTTCCATCTGTCC<br>ATACCCAACTTCAATCAGTACGAAGCCATGAG<br>TTGTGATTTTAATGGAGGGAAAATTTCAATACA<br>ATACAATTTGAGCCACAGCACAGTCACCGACT<br>CAGCAAATCACTGCGGGACAGTTGCCAATGGC<br>ATCCTAGAAACTTTCCACAAATTT	186	9-24 (S)<br>(440-625)	91.4
10	AAGTTAATTGATGTAATGATGAGTAAGATTGA<br>TGCAAGGAAATTTGAGAATGAAGTCTGGGATG<br>ATCTAAAAACGCTATGCAGCATGCACACTGGG<br>GTGGTTGTTGAAAAGAAGAAGAGAGGTGGCAA<br>GCAGGAAATAACACCTCACTGTGCACTTTTGG<br>ATTGCATCATGTACGAG	177	9-24 (S)<br>(1687-1863)	90.8
11	ATGACTGGTATGGACCCGACAAGCAAGACCTG<br>GATTGATATTGAAGGTAGGGCTGAAGATCCAG<br>TGGAGATTGCCATCTACCAGCCTGCTGGTGGA<br>CAGTACATCCATTTCTACAGAGAGCCAACAGA<br>CGCCAAGCAGTTCAAGCAAGACTCTAAATATT<br>CACATGGTATTGACATTGTTGACCTATTTAAA	192	9-24 (S)<br>(1981-2172)	95.8
12	CTAAATCAAGCAGTCAACAACCTTGTTGAGTT<br>AAAATCTCAACAACAGAAAAATGTGCTGAGTG<br>TGGGGCAACTGTCATCTGACGACCTCCTAATTC<br>TTGCTGCTGACATTGACAGACTGAAGGCAAAA<br>ATAACCAGGACGGAGAGACCTCAATCCAATGG<br>GGTCTACATGGGGATATCTGCAGAGCTCCGG	192	9-24 (S)<br>(2901-3087)	93.1

Supplementary Table 2. The primers used for amplifying the complete genome of WENV

Segment	Primers	Sequences (5'-3')
	WENV-S-F_{1}	TTAGGGGATCCTAGGGGTT
	WENV-S-F_{34}	TAGAAGAGACAAAGTGATGGG
	WENV-S-F_{871}	TCTTCCTGGTGGTTATTGTT
**S segment**	WENV-S-F_{2942}	CTCTCCGTCCTGGTTATCT
	WENV-S-R_{1195}	GCTGGTTCGGTTACTTCT
	WENV-S-R_{3128}	AAGACAAGAGGAACGATGG
	WENV-S-R_{3312}	GCTTACTACTGTGGCAATATG
	WENV-L-F_{1}	GGACCTAGGCATTTTGATC
	WENV-L-F_{15}	TGATCTATC ATGGAAGARAYTC
	WENV-L-R_{1536}	GAGACGACAGAACCTTGAA
	WENV-L-F_{202}	GYRACCAYAAYTCTGAATCC
	WENV-L-F_{835}	CRGAATTGTATGAGTCAGAAC
	WENV-L-R_{2168}	TGTTATCAAGTGGCAGAAGT
**L segment**	WENV-L-F_{1290}	ACGCCAACACTCAATCAA
	WENV-L-F_{1916}	GTGTCAACCGAGTAAGAGAT
	WENV-L-R_{3731}	CCAGCAGAGTAATGTTGAGA
	WENV-L-F_{3303}	GCAGCCTTGAAGAAYCTRT
	WENV-L-F_{3340}	TCRCCAGAGTCWTTCACAT
	WENV-L-R_{5892}	GCAGGTGTATATGGAAGTTCT
	WENV-L-F_{5683}	TWGGYCCRCTKTCATCAT
	WENV-L-R_{6307}	TAACCATCCTTCAGCACAA
	WENV-L-R_{7106}	CGACTTATTTCTGAGTACATGG

Supplementary Table 3. **The reference sequences used in this study.**

**Strains**	**GenBank accession no. L**	**GenBank accession no. S**	**Complete genome or partial gene**	**Sampling sites**
WENV 9-24	MF414207.1	MF414208.1	Complete genome	China
WENV Rn366	KM386661.1	KM386660.1	Complete genome	Zhejiang Province, China
WENV Rn242	NC_026019.1	NC_026018.1	Complete genome	Zhejiang Province, China
WENV HUM	MF595888.1	MF595889.1	Complete genome	Haikou, Hainan Province, China
WENV PL/DK	MF974577.1	MF974578.1	Complete genome	Haikou, Hainan Province, China
WENV G107	MF925714.1	MF925715.1	Complete genome	Shandong Province, China
WENV MYR039	MG999644.1	MG999643.1	Complete genome	Borneo, Malaysia
WENV RnYCB1	KY662262.1	KY662263.1	Complete genome	Xinjiang Province, China
Cardamones C649	KC669690.1	KC669696.1	Complete genome	Cambodia
Cardamones C617	KC669691.1	KC669694.1	Complete genome	Cambodia
WZ140510	KM051421.1	KM051423.1	Complete genome	Zhejiang Province, China
WZ140512	KM051420.1	KM051422.1	Complete genome	Zhejiang Province, China
RatArenavirus RnYL4-2016	MG736231.1	MG736235.1	Complete genome	Yunnan Province, China
RatArenavirus RtYM16-2015	MG736227.1	MG736236.1	Complete genome	Yunnan Province, China
RatArenavirus RnYM3-2016	MG736229.1	MG736233.1	Complete genome	Yunnan Province, China
RatArenavirus RnYM51-2016	MG736230.1	MG736234.1	Complete genome	Yunnan Province, China
RatArenavirus RtYM51-2015	MG736228.1	MG736232.1	Complete genome	Yunnan Province, China
RatArenavirus 2015SZCDC64	KY659346.1	/	Partial gene	Shenzhen, Guangdong Province, China
RatArenavirus 2015SZCDC41	KY659338.1	/	Partial gene	Shenzhen, Guangdong Province, China
RatArenavirus 2015SZCDC16	KY659339.1	/	Partial gene	Shenzhen, Guangdong Province, China
RatArenavirus 2013SZ18	KY659345.1	/	Partial gene	Shenzhen, Guangdong Province, China
RatArenavirus 2013SZ28	KY659344.1	/	Partial gene	Shenzhen, Guangdong Province, China
RatArenavirus 2013SZ16	KY659343.1	/	Partial gene	Shenzhen, Guangdong Province, China
RatArenavirus 2013SZ17	KY659342.1	/	Partial gene	Shenzhen, Guangdong Province, China
RatArenavirus 2013SZ24	KY659341.1	/	Partial gene	Shenzhen, Guangdong Province, China
RatArenavirus 2013SZ34	KY659340.1	/	Partial gene	Shenzhen, Guangdong Province, China
RatArenavirus 2016SZ166	KY659337.1	/	Partial gene	Shenzhen, Guangdong Province, China
RatArenavirus 2016SZ167	KY659336.1	/	Partial gene	Shenzhen, Guangdong Province, China
RatArenavirus 2016SZ170	KY659335.1	/	Partial gene	Shenzhen, Guangdong Province, China
RatArenavirus 2016SZ132	KY659334.1	/	Partial gene	Shenzhen, Guangdong Province, China
RatArenavirus 2016SZ150	KY659333.1	/	Partial gene	Shenzhen, Guangdong Province, China

Supplementary Table 4. The genome organization of Wenzhou virus strains identified in Guangzhou*

WENV Strains	L	RdRp		IGR	ZP		S	GPC		IGR	NP	
	nt	nt	aa		nt	aa	nt	nt	aa		nt	aa
RnGZ37-2018	7118	6672	2223	122	276	91	3350	1479	492	62	1704	567
RnGZ40-2018	7118	6672	2223	122	276	91	3350	1479	492	62	1704	567
Cardamones C649	7178	6687	2228	110	276	91	3329	1479	492	62	1704	567
Cardamones C617	7171	6687	2228	109	276	91	3344	1479	492	62	1704	567
9-24	7113	6669	2222	120	276	91	3350	1479	492	62	1704	567
Rn366	7113	6669	2222	120	276	91	3350	1479	492	62	1704	567
Rn242	7146	6687	2228	109	276	91	3337	1479	492	62	1704	567
HUM	7147	6693	2230	109	276	91	3334	1479	492	62	1704	567
PL/DK	7147	6693	2230	109	276	91	3338	1479	492	62	1704	567
G107	7131	6687	2228	108	276	91	3322	1479	492	62	1704	567
MYR039	7159	6687	2228	108	276	91	3345	1479	492	62	1704	567
RnYCB1	7164	6687	2228	110	276	91	3343	1479	492	62	1704	567
WZ140510	7090	6684	2227	225	156	91	3276	1425	492	62	1716	571
WZ140512	6949	6687	2228	146	105	91	3261	1425	492	62	1704	567
RnYL4-2016	7151	6687	2228	111	276	91	3084	1479	492	62	> 1495	> 497
RtYM16-2015	7153	6687	2228	110	276	91	3351	1479	492	62	1704	567
RnYM3-2016	7159	6687	2228	110	276	91	3328	1479	492	62	1704	567
RnYM51-2016	7152	6687	2228	111	276	91	3318	1479	492	62	1704	568
RtYM51-2015	7140	6687	2228	109	276	91	3377	1479	492	62	1704	568

*L, large segment; RdRp, RNA-dependent RNA polymerase; ZP, zinc binding matrix protein; S, small segment; GPC, glycoprotein precursor;NP, nucleoprotein; IGR, intergenic region; nt, nucleotide; aa, amino acid.

Supplementary Table 5. WENV recombination events detected using the RDP package

Segment	Recombinant strain (GenBank accession no.)	Major pwerent strain (GenBank accession no.)	Minor pwerent strain (GenBank accession no.)	RDPRCS	Tools (Methods with significant *P*-value for this recombination)
L	WZ140510	WZ140512	Unknown	0.642	RDP, MaxChi, Chimaera, 3Seq, BootScan
S	RnYM3-2016	RtYM51-2015	RnYL4-2016	0.607	RDP, GENECONV, MaxChi, Siscan, 3Seq, BootScan
	Rn242	DK	WZ140510	0.676	RDP, GENECONV, MaxChi, Chimaera, Siscan, 3Seq, BootScan

Minor pwerent: Pwerent contributing the smaller fraction of sequence; Major pwerent: Pwerent contributing the larger fraction of sequence; Unknown: Only one pwerent and a recombinant need be in the alignment for a recombination event to be detectable. The sequence listed as unknown was used to infer the existance of a missing pwerental sequence. RDPRCS: The RDP recombination consensus score.

A confirmed recombination event was required to satisfy the following two conditions (as suggested by the RDP manual): i) the event could be verified by at least two methods with $P < 0.05$; and ii) the RDP recombination consensus score (RDPRCS) was $N > 0.60$. If an event met the first condition, but had an RDPRCS in the range 0.4-0.6, it was considered a possible recombination; otherwise the event was rejected.

Supplementary Figures

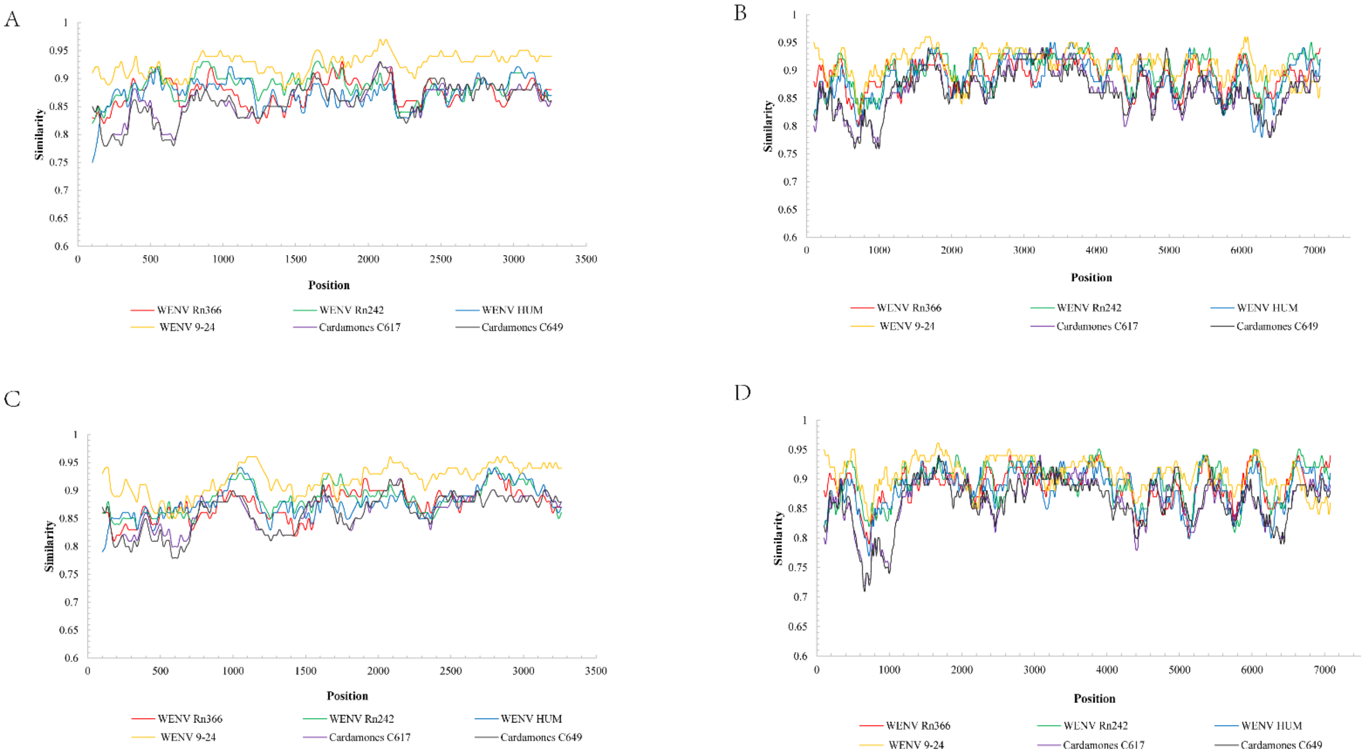

Supplementary Figure 1. The recombination analysis of RnGZ37-2018 and RnGZ40-2018 by similarity plots.

The recombination events were analyzed based on the full-length of L/S from RnGZ37-2018, RnGZ40-2018 and other representative WENV strains. RnGZ37-2018 and RnGZ40-2018 were as query sequence. The recombination analysis: (A) S segment of RnGZ37-2018; (B) L segment of RnGZ37-2018; (C) S segment of RnGZ40-2018; (D) L segment of RnGZ40-2018.

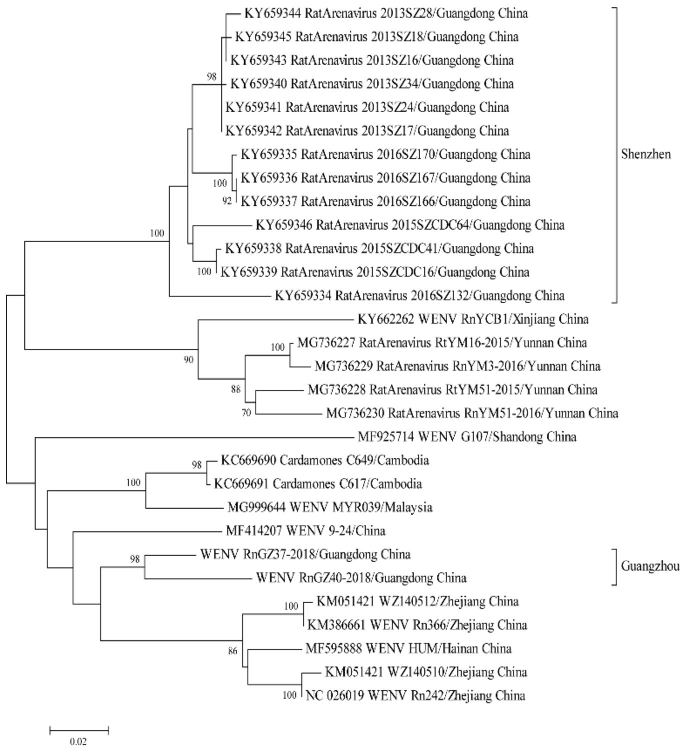

Supplementary Figure 2. Phylogenetic analysis of the partial RdRp gene (589 bp) from WENV in Guangzhou and Shenzhen, China.

The phylogenetic tree was performed using ML methods based on GTR model with gamma distribution and invariant sites, and evaluated with 1000 bootstrap replicates. Sequences were identified by the GenBank accession number and the strain name, followed by their origin. The partial sequences of WENV isolated from Shenzhen and Guangzhou were indicated.
